# Supplementary material for: End Sequence Analysis Toolkit (ESAT) expands the extractable information from single-cell RNA-seq data
Source: Genome Res. 2016 Oct;26(10):1397–410. doi: 10.1101/gr.207902.116 (PMC5052061; doi:10.1101/gr.207902.116)
Supplement: Supplemental Material [file supp_gr.207902.116_Supp_Materials.tar.gz › ESAT-esat_v0.1/doc/html/classbroad_1_1pda_1_1geneexpression_1_1agilent_1_1_agilent_utils.html]

ESAT: broad.pda.geneexpression.agilent.AgilentUtils Class Reference


ESAT


- Main Page
- Related Pages
- Packages
- Classes
- Files

- Class List
- Class Index
- Class Hierarchy
- Class Members

All Classes Namespaces Files Functions Variables Enumerator Pages

- broad
- pda
- geneexpression
- agilent
- AgilentUtils

Static Public Member Functions |
List of all members

broad.pda.geneexpression.agilent.AgilentUtils Class Reference

|  |  |
| --- | --- |
| Static Public Member Functions | |
| static Map< String, AgilentProbe > | parseAgilentExperiment (File file) throws IOException |
|  | |
| static AgilentArrayStats | parseAgilentExperimentGlobalStats (File file) throws IOException |
|  | |
| static Map< String,   ExpressionExperimentInfo > | parseExperimentInfoFile (File experimentInfoFile) throws IOException |
|  | |
| static Map< String, String > | parseExperimentInfoFileToName (File experimentInfoFile) throws IOException |
|  | |
| static Map< String, String > | parseExperimentInfoFileToName (File experimentInfoFile, boolean passingFilter) throws IOException |
|  | |
| static Map< String, Collection  < ExpressionExperimentInfo > > | parseExperimentInfoFileToGroup (File experimentInfoFile) throws IOException |
|  | |
| static Map< String, Collection  < String > > | parseExperimentInfoFileToGroups (File experimentInfoFile) throws IOException |
|  | |
| static Map< String, Double > | makeDataMatrix (File agilentFile, Collection< String > nonFlaggedGenes) throws IOException |
|  | |
| static MatrixWithHeaders | makeDataMatrix (File[] agilentFiles) throws IOException |
|  | |
| static MatrixWithHeaders | makeDataMatrix (File[] agilentFiles, Map< String, ExpressionExperimentInfo > experimentInfo) throws IOException |
|  | |
| static Map< String, Collection  < String > > | parseExperimentInfoGroups (String experimentInfoFile) throws IOException |
|  | |
| static Map< String, Collection  < String > > | parseExperimentInfoNonControls (String experimentInfoFile) throws IOException |
|  | |
| static Collection< String > | parseControlSamples (String experimentInfoFile) throws IOException |
|  | |

## Member Function Documentation

|  |  |  |  |
| --- | --- | --- | --- |
| static Map<String, Double> broad.pda.geneexpression.agilent.AgilentUtils.makeDataMatrix | ( | File | *agilentFile*, |
|  |  | Collection< String > | *nonFlaggedGenes* |
|  | ) |  | throws IOException |

static

Here is the call graph for this function:

Here is the caller graph for this function:

|  |  |  |  |  |  |
| --- | --- | --- | --- | --- | --- |
| static MatrixWithHeaders broad.pda.geneexpression.agilent.AgilentUtils.makeDataMatrix | ( | File[] | *agilentFiles* | ) | throws IOException |

static

Here is the call graph for this function:

|  |  |  |  |
| --- | --- | --- | --- |
| static MatrixWithHeaders broad.pda.geneexpression.agilent.AgilentUtils.makeDataMatrix | ( | File[] | *agilentFiles*, |
|  |  | Map< String, ExpressionExperimentInfo > | *experimentInfo* |
|  | ) |  | throws IOException |

static

Here is the call graph for this function:

|  |  |  |  |  |  |
| --- | --- | --- | --- | --- | --- |
| static Map<String, AgilentProbe> broad.pda.geneexpression.agilent.AgilentUtils.parseAgilentExperiment | ( | File | *file* | ) | throws IOException |

static

|  |  |  |  |  |  |
| --- | --- | --- | --- | --- | --- |
| static AgilentArrayStats broad.pda.geneexpression.agilent.AgilentUtils.parseAgilentExperimentGlobalStats | ( | File | *file* | ) | throws IOException |

static

Here is the caller graph for this function:

|  |  |  |  |  |  |
| --- | --- | --- | --- | --- | --- |
| static Collection<String> broad.pda.geneexpression.agilent.AgilentUtils.parseControlSamples | ( | String | *experimentInfoFile* | ) | throws IOException |

static

|  |  |  |  |  |  |
| --- | --- | --- | --- | --- | --- |
| static Map<String, ExpressionExperimentInfo> broad.pda.geneexpression.agilent.AgilentUtils.parseExperimentInfoFile | ( | File | *experimentInfoFile* | ) | throws IOException |

static

|  |  |  |  |  |  |
| --- | --- | --- | --- | --- | --- |
| static Map<String, Collection<ExpressionExperimentInfo> > broad.pda.geneexpression.agilent.AgilentUtils.parseExperimentInfoFileToGroup | ( | File | *experimentInfoFile* | ) | throws IOException |

static

Here is the call graph for this function:

|  |  |  |  |  |  |
| --- | --- | --- | --- | --- | --- |
| static Map<String, Collection<String> > broad.pda.geneexpression.agilent.AgilentUtils.parseExperimentInfoFileToGroups | ( | File | *experimentInfoFile* | ) | throws IOException |

static

Here is the call graph for this function:

|  |  |  |  |  |  |
| --- | --- | --- | --- | --- | --- |
| static Map<String, String> broad.pda.geneexpression.agilent.AgilentUtils.parseExperimentInfoFileToName | ( | File | *experimentInfoFile* | ) | throws IOException |

static

|  |  |  |  |
| --- | --- | --- | --- |
| static Map<String, String> broad.pda.geneexpression.agilent.AgilentUtils.parseExperimentInfoFileToName | ( | File | *experimentInfoFile*, |
|  |  | boolean | *passingFilter* |
|  | ) |  | throws IOException |

static

Here is the call graph for this function:

|  |  |  |  |  |  |
| --- | --- | --- | --- | --- | --- |
| static Map<String, Collection<String> > broad.pda.geneexpression.agilent.AgilentUtils.parseExperimentInfoGroups | ( | String | *experimentInfoFile* | ) | throws IOException |

static

|  |  |  |  |  |  |
| --- | --- | --- | --- | --- | --- |
| static Map<String, Collection<String> > broad.pda.geneexpression.agilent.AgilentUtils.parseExperimentInfoNonControls | ( | String | *experimentInfoFile* | ) | throws IOException |

static

---

The documentation for this class was generated from the following file:

- java/broad/pda/geneexpression/agilent/AgilentUtils.java


---

Generated on Fri Aug 15 2014 15:11:00 for ESAT by  

 1.8.7
